# Participation of Amyloid and Tau Protein in Neuronal Death and Neurodegeneration after Brain Ischemia

**DOI:** 10.3390/ijms21134599

**Published:** 2020-06-28

**Authors:** Ryszard Pluta, Marzena Ułamek-Kozioł, Sławomir Januszewski, Stanisław J. Czuczwar

**Affiliations:** 1 Laboratory of Ischemic and Neurodegenerative Brain Research, Mossakowski Medical Research Centre, Polish Academy of Sciences, 02-106 Warsaw, Poland; mulamek@imdik.pan.pl (M.U.-K.); sjanuszewski@imdik.pan.pl (S.J.); 2 Department of Pathophysiology, Medical University of Lublin, 20-090 Lublin, Poland; czuczwarsj@yahoo.com

**Keywords:** brain ischemia, amyloid, tau protein, secretases, presenilin, neuronal death, neurodegeneration, amyloid plaques, neurofibrillary tangles, dementia, genes

## Abstract

Current evidence indicates that postischemic brain injury is associated with the accumulation of folding proteins, such as amyloid and tau protein, in the intra- and extracellular spaces of neuronal cells. In this review, we summarize protein changes associated with Alzheimer’s disease and their gene expression (amyloid protein precursor and tau protein) after brain ischemia, and their roles in the postischemic period. Recent advances in understanding the postischemic mechanisms in development of neurodegeneration have revealed dysregulation of amyloid protein precursor, α-, β- and γ-secretase and tau protein genes. Reduced expression of the α-secretase gene after brain ischemia with recirculation causes neuronal cells to be less resistant to injury. We present the latest data that Alzheimer’s disease-related proteins and their genes play a crucial role in postischemic neurodegeneration. Understanding the underlying processes of linking Alzheimer’s disease-related proteins and their genes in development of postischemic neurodegeneration will provide the most significant goals to date for therapeutic development.

## 1. Introduction

Brain ischemia is one of the most common forms of neurodegeneration, with a series of pathological molecular processes that occur during and after ischemia and gradually spread to various brain structures. New data suggest that there is a similarity between neuropathology developed in the postischemic brain and Alzheimer’s disease [1,2,3,4,5,6,7,8]. Both ischemic stroke in human and experimental ischemic brain episodes are life-threatening pathological events with development of Alzheimer’s disease-type dementia after ischemia [9,10,11,12,13,14,15]. Current studies indicate that ischemia-reperfusion brain injury can be involved in development of Alzheimer’s disease neuropathology [3,16]. First, ischemic stroke and Alzheimer’s disease have the same risk factors like age, hyperlipidemia, hypertension and diabetes. Second, the postischemic brain generates a unique pattern of disappearance of neuronal cells in the CA1 area of the hippocampus with serious general brain atrophy, which is similar to the atrophy noted in Alzheimer’s disease [17,18,19,20,21]. Third, neuroinflammatory reactions have an important role in the progress of the postischemic brain and Alzheimer’s disease [22,23]. Fourth, the data suggest that postischemic brain injury with recirculation can trigger the neuropathology of folding proteins characteristic of Alzheimer’s disease by generation and accumulation of amyloid [19,24,25,26]. Finally, investigations proved that tau protein pathology also played a significant role in progression after ischemic brain neurodegeneration [27,28,29,30,31,32,33,34,35,36,37,38]. In this review, we present changes in expression of genes involved in the amyloidogenic metabolism of the amyloid protein precursor, which are associated with the production of amyloid in the postischemic brain. In addition, we pay attention to whether the amyloid is involved in death of neurons in the CA1 and CA3 areas of the hippocampus and the medial temporal cortex postischemia. Also, we take into account the importance of ischemic changes in gene expression of tau protein during clinical onset, progression and maturation of brain neurodegeneration after ischemia. Below we summarize the latest data that Alzheimer’s disease-related proteins, like amyloid and tau protein, and their genes, play a fundamental role in postischemic neurodegeneration. Progress in understanding new key processes induced by brain ischemia with recirculation, like changes in the genotype and phenotype of the Alzheimer’s disease type, which are not yet fully explained, may help develop strategies for prevention and treatment against neurodegeneration induced by ischemia.

## 2. Dysregulation of Amyloid Processing Genes in Global Cerebral Ischemia due to Cardiac Arrest in Rats

In the CA1 area of the hippocampus, gene expression of the amyloid protein precursor was below the control value within 2 days after ischemia and 7 and 30 days postischemia the expression of the amyloid protein precursor gene was above the control values (Table 1) [39]. In the CA3 region, 2, 7 and 30 days following ischemia, the expression of the amyloid protein precursor gene was above the control values (Table 1) [38]. In the medial temporal cortex, gene expression of the amyloid protein precursor was below the control value within 2 days after ischemia and 7 and 30 days postischemia the expression of the amyloid protein precursor gene was above the control values (Table 1) [40].

The expression of the β-secretase gene went above the control values in the hippocampal CA1 area 2–7 days postischemia but 30 days following ischemia the β-secretase gene expression was below the control value (Table 2) [39]. The expression of the β-secretase gene was below the control values in the hippocampal CA3 area 2–7 days following ischemia. On the contrary, 30 days postischemia, β-secretase gene expression was above control (Table 2) [38]. The expression of the β-secretase gene was above the control value in the medial temporal cortex 2 days postischemia but 7–30 days postischemia gene expression was reduced (Table 2) [40]. 

In the CA1 region of the hippocampus, expression of the presenilin 1 gene increased 2–7 days after ischemia but 30 days postischemia the expression of this gene was below the control values (Table 3) [39]. In the CA3 area of the hippocampus, expression of the presenilin 1 gene increased 2–7 days postischemia, and was below the control values 30 days after ischemia (Table 3) [38]. In the medial temporal cortex, the expression of the presenilin 1 gene oscillated around the control values during 2, 7 and 30 days postischemia (Table 3) [41].

In the CA1 area of the hippocampus, expression of the presenilin 2 gene increased 2–7 days postischemia but, in contrast, 30 days following ischemia the expression of this gene was below the control values (Table 4) [39]. In the CA3 field of the hippocampus, expression of the presenilin 2 gene decreased 2–7 days after ischemia. On the contrary, 30 days postischemia, the expression of this gene increased above the control values (Table 4) [38]. In the medial temporal cortex, the expression of the presenilin 2 gene was above the control value on day 2 postischemia (Table 4) [41]. In contrast, the expression of this gene oscillated around the control values during 7–30 days postischemia (Table 4) [41]. 

In the CA3 region 2, 7 and 30 days postischemia, the expression of the α-secretase gene was below the control values (Table 5) [38]. 

## 3. Amyloid Staining in Experimental Postischemic Brain 

Postischemic brain injury, with a survival period up to 2 years, revealed brain parenchyma staining to amyloid. The staining was found in intra- and extracellular spaces [18,21,24,42,43,44,45,46,47,48,49,50,51,52,53,54,55]. Amyloid was noted in neurons and neuroglial cells [18,47,50,56,57,58,59,60]. Astrocytes with massive accumulation of amyloid might be involved in the development of glial scar [18,50,58,59,60]. Additionally, reactive astrocytes with huge accumulation of amyloid might be involved in repair of postischemic tissue with associated astrocyte death [18,24,50,61,62].

Staining for amyloid has been noted in subcortical white matter and the periventricular area postischemia [19,63,64]. The more intense injury of postischemic white matter is, the more widespread staining of amyloid in the brain parenchyma occurs [65]. It is assumed that the above kind of alterations are responsible for the development of leukoaraiosis after an ischemic brain episode [64]. Extracellular deposits of amyloid ranged from very small dots to typical diffuse amyloid plaques [18,19,20,24,48,50,66,67,68,69,70,71]. Multifocal diffuse amyloid plaques were observed in the ischemic hippocampus, cortex, corpus callosum and around the lateral ventricles. The accumulation of diffuse amyloid plaques in response to ischemia-reperfusion brain injury in rats was not transient, since it has been documented that these plaques transform into senile amyloid plaques during one year after an ischemic episode [72]. 

The accumulation of amyloid inside neuronal cells and astrocytes underscores the possible importance of amyloid in the occurrence of postischemic neurodegeneration [24,45,59,60,67,68]. In addition, these accumulations may influence synaptic disintegration and turn on further retrograde neuronal death after ischemia. These facts indicate that gradual postischemic amyloid accumulation may be responsible for additional neurodegenerative mechanisms that could worsen the outcome during recirculation by continuous neuronal death [9,19,21,47,48,54,55,73,74,75]. Postischemia amyloid is generated as a product of neuronal death [44] and as a final point shows its own neurotoxic effects. Amyloid is a neurotoxic particle and triggers intracellular mechanisms in neurons and neuroglial cells that cause additional neuronal and neuroglial cell injury or death after ischemia [55,76].

## 4. Dysregulation of Tau Protein Gene in Global Cerebral Ischemia due to Cardiac Arrest in Rats

In the CA1 field of the hippocampus, gene expression of the tau protein was above the control value within 2 days postischemia and 7–30 days postischemia the expression of the above gene was below the control values (Table 6) [8,37]. In the CA3 area of hippocampus, its expression was opposite (Table 6) [8,38]. 

## 5. Tau Protein Staining in Experimental Postischemic Brain

Massive staining of tau protein at neurons was found in the ischemic hippocampus and brain cortex [52,77,78]. Tau protein staining was also noted in astrocytes, microglia and oligodendrocytes, postischemia [31,33,79,80,81]. The above observations indicated that neuronal and neuroglial cells display abnormalities in tau protein after an ischemic brain episode [79], which may illustrate a prime pathological stage of the ischemic processes in these cells [80]. Another study showed that tau protein itself can inhibit the transport of amyloid in the way of the neuron body at axons and dendrites, leading to amyloid accumulation in the body of neurons [82]. Available evidence shows that postischemia, hyperphosphorylated tau protein dominates in neurons and goes along with apoptosis [28,31,33,83,84]. The above-mentioned data indicate that neuronal apoptosis after ischemic brain injury is straightway connected with tau protein hyperphosphorylation. Other observations revealed that transient brain ischemia was engaged in a neurofibrillary tangle-like formation postischemia [28,83,84]. The above data provide a neuropathological basis for neurodegeneration following brain ischemia with recirculation [28]. 

## 6. Amyloid and Tau Protein in Postischemic Human Brain and Plasma

In autopsy studies of human postischemic brains, the relationship between ischemia and the accumulation of amyloid was observed [25,85,86,87]. Studies have documented diffuse and senile amyloid plaques in the hippocampus and cortex [25,85,86,87]. The neurons’ staining for amyloid depended on the brain area. Cortical and hippocampal neurons were the most intensely stained. In contrast, the staining of dentate gyrus neuronal cells appeared to be weak. Some neuronal cells were also labeled with antibodies against tau-1 [86]. Ependymal and epithelial cells were stained intensively for amyloid. Examined brain vessels of gray and white matter were surrounded by amyloid deposits. The deposits had mainly a cuffs shape. In postischemic brains, weak staining for amyloid was noted around blood–brain barrier vessels [86]. Amyloid around the blood–brain barrier microvessels indicated that it originated from serum. Data from a clinical study showing that plasma amyloid had been raised in patients following brain ischemia supported the above suggestion [26,88,89]. According to another study, β-amyloid peptide 1–40 and 1–42 were documented in the human postischemic hippocampus [25]. This strong staining of different amyloid forms may contribute to the development of postischemic neurodegeneration. 

Observations of patients for a period of 4 days postischemia showed an increase in blood amyloid [26]. The increase correlated with clinical outcome after ischemic brain injury [26]. The results support the notion that ischemic brain with reperfusion may play a key role in the amyloidogenic processing of amyloid protein precursor. Tau protein was evident in plasma postischemia in humans with two peaks at 2 and 4 days, and most likely indicated the progression of neuron changes during recirculation [29]. Observed bimodal elevation of tau protein in blood is consistent with two types of neuronal death: firstly via necrosis and secondly by apoptosis [30]. It seems likely that the profiles reflect a time course of primary and secondary postischemic neuronal alterations [30]. The above observations suggest that tau protein in human blood has the potential to be used as a predictor for the neurological outcome postischemia [29,30]. Other data revealed that transient focal cerebral ischemia in humans was involved in the development of neurofibrillary tangles [27].

## 7. Neuropathophysiology in Postischemic Brain

After ischemia with reperfusion, a massive release of excitatory amino acids, and an intracellular overload of calcium in the hippocampus, were documented [90]. The release of glutamate from presynaptic endings, and its deficient reuptake, triggered an increase of glutamate in the extracellular space of the hippocampus [90]. As a consequence of the above-mentioned process, glutamate receptors were excessively stimulated, leading to an enormous influx of calcium to the neuronal cells by calcium channels [90]. Consequently, calcium was released from intracellular compartments into the neuronal cytoplasm. Subsequently, intracellular calcium could activate miscellaneous enzymes, necessary for survival or death of neurons. For instance phospholipases, endonucleases, nitric oxide synthase and proteases are activated via calcium and, as an ending effect of this course of action, injury to the nucleus, membranes and cytoplasm organelles, with loss of neuronal cells, was observed. The above-mentioned pathological pathways play a part in neuronal damage or death postischemia. As a rule, neuronal death after an ischemic brain episode was considered as necrotic, but eventually the neuronal cells die as a result of apoptosis. Necrosis happens due to deficit of energy and abnormal osmotic homeostasis and involves a huge number of neurons in the brain parenchyma. The postischemic neurons swell as they take up an overload of water and rupture of the cytoplasmic membrane takes place, which results in an outflow of neuronal contents into the neighboring tissue. DNA cleavage in necrotic neurons is a delayed phase occurring by mechanisms needing serine proteases. The quick drop of energy in neurons, and glucose uptake postischemia, are responsible for necrosis. Apoptosis has been noted in neurons of the CA1 region of the hippocampus 4 days after brain ischemia with reperfusion. Postischemia, caspase-3 plays a key role in the death of neurons [91,92,93,94]. The connection of autophagy and mitophagy with apoptosis should be suggested, too [91,92,93,94]. Delayed neuronal death after postischemic injury is controlled by apoptotic routes. Presently, one more neuronal cell death pathway called necroptosis was documented after brain ischemia with reperfusion. In this mechanism, postischemic neurons exhibited features of both necrotic and apoptotic processes. Another process of neuronal death is called autophagic-programmed cell death. In this event, autophagosomes and autolysosomes are found in dying neuronal cells. Recent evidence indicated that autophagy and mitophagy play a significant role in postischemic brain injury [91,92,93,94].

## 8. Neuropathology in Postischemic Brain 

The death of neurons in the CA1 hippocampal area develops 2–7 days following ischemia and is called delayed neuronal death. Extending survival following brain ischemia injury, e.g., up to 2 years, triggers alterations in neuronal cells in the hippocampal areas with nonselective sensitivity to ischemia, e.g., in the CA3 area [19]. In contrast, changes in the striatum, mainly of medium-sized neurons, are noted primarily in the dorsolateral area. In the postischemic cortex, layers 3, 5 and 6 presented numerous neuronal changes [18,67,68]. Borderline zones of the brain cortex were also a region of intense neuronal changes after ischemia. Between 6–24 months following ischemia insult of brain, in addition to localized neuronal death various types of pathological neuronal injuries were observed. The first took the form of chronic neuronal change. Other changes were acute neuronal modifications postischemia and were present in those regions of brain parenchyma which were not involved in early changes, e.g., sectors CA2, CA3 and CA4 of the hippocampus [19]. Disappearance of neuronal cells in the CA1 hippocampal area, with a decrease in acetylcholine level, was found following focal brain ischemia. This suggests that neuronal death may also result from failure of neuronal stimulation and cholinergic transmission [90]. 

In the regions of massive neuronal damage, an intense response of microglia and astrocytes was observed [18,22,23,67,68,95]. Additionally, postischemic astrocytes in the hippocampal CA1 region showed an enhanced response to cytokines [95]. This evidence indicates that the rise in neuroinflammatory mediators in astrocytes is directly related to the selective sensitivity of neurons to brain injury as a result of an ischemic episode [95]. The above data imply that neuronal cells in sensitive regions of the ischemic brain are targets for interleukin-1β produced by astrocytes. This is explained by the intensified expression of the neural interleukin-1 receptor. Also, interleukin-1β has been proved to play an important role in the development of alterations in brain cells and the development of edema after brain injury due to an ischemic episode with reperfusion. Postischemic inflammatory mediators can trigger a self-sustaining cycle that leads postischemic pathology to neurodegeneration. In brain ischemia, interleukin-1 is a key player stimulating neurons to amyloidogenic processing of the amyloid protein precursor along with the release of neuroinflammatory mediators. These processes cause abnormality in the functioning of neurons and, in the end, their death with irreversible disruption of the neural network. The death of neurons results from, among others causes, neuroinflammatory mediators, which additionally cause neuronal pathology. This process activates microglia causing further strengthening, which leads to self-propagation of the inflammatory cycle. Moreover, strong evidence was provided that the amyloid which is produced following brain ischemia [19,24,50] promotes the release of inflammatory mediators by microglia. In the hippocampus, activation of neuroglial cells precedes neuronal injury and neuronal death, and lasts long after an ischemic episode [22,23]. 

There is plenty evidence of abnormal synaptic activity following experimental brain ischemia [96,97]. This is supported by ultrastructural observations in the hippocampal CA1 region following ischemia [97]. Other studies have revealed that brain ischemia triggers the activity of synaptic autophagy, which is associated with the death of neurons in the hippocampal CA1 area following transient ischemia [98,99]. What is more, a reduction in excitatory synaptic transmission was noted after brain ischemia in the hippocampal CA1 subfield [90]. The rise in intracellular calcium following an ischemic episode activates calpain activity in neurons, and calpain target proteins are present in glutaminergic and GABAergic synapses. In ischemia-reperfusion brain injury, calpain cleaves pre- and postsynaptic proteins. Calpain-related cleavage of proteins contributes to the loss of neuronal cells in ischemic brain parenchyma [100].

The effect of brain ischemia on the permeability of the blood-brain barrier has been widely studied for many years, and vice versa. Ischemia-reperfusion brain injury triggers a number of changes that increase the permeability of the blood-brain barrier to cellular and noncellular blood elements (e.g., platelets, amyloid) and lead to opening of tight junctions and diffuse leakage of blood elements through the necrotic wall of the blood vessel [63,70,101,102,103,104]. In postischemic damage to the blood-brain barrier, two unusual features deserve attention. One is important because of the chronic effects of extravasated amyloid [105,106] in the development of neurodegeneration, and the other relates to the leakage of platelets, which causes massive toxic, mechanical and rapid destruction of brain parenchyma [107]. It has long been known that platelets continuously produce a neurotoxic amyloid. The ability of the amyloid to cross the ischemic blood-brain barrier leads to its location of neurotoxic effects on specific neuronal populations, which may then lead to subsequent increased production of amyloid in brain parenchyma. Circulating amyloid is delivered to ischemic brain tissue and, therefore, contributes to brain amyloidosis, vasoconstriction and the development of cerebral amyloid angiopathy following an ischemic-reperfusion brain episode [25,26,28,85,86,87,104,105,108,109]. 

Animal and human brains respond to brain ischemia by inducing neuroinflammation [22,23,110]. Shortly following brain ischemia-reperfusion injury, neurons and neuroglial cells trigger molecular reactions that lead to the activation of astrocytes up to 28 days after ischemia. Activated astrocytes multiply quickly and change their shape and function [110]. After activation, astrocytes secrete proinflammatory cytokines, metalloproteinases and chemokines [110]. Substances released from astrocytes, e.g., interleukin-1β and matrix metalloproteinases, increase the permeability of the blood-brain barrier and enhance the transfer of leukocytes from the blood to the brain parenchyma [110]. The influx of these cells leads to further progressive ischemic tissue injury [22,23]. Microglia, like astrocytes, also belong to the first line of defense and are activated within a few minutes following brain ischemia [110]. Increased activation is noted 2–3 days after the onset of ischemia, persisting for several weeks postischemia [110]. Activated microglial cells change their shape to amoeboid and thus acquire phagocytic capacity [110]. Microglia secrete proinflammatory substances such as matrix metalloproteinase-9, tumor necrosis factor α and interleukin-1, which are involved in blood-brain barrier injury. Intensified influx of monocytes into ischemic brain parenchyma is observed during 24 h postischemia as a result of additional damage to the blood-brain barrier by astrocytic and microglial inflammatory mediators. An increased number of monocytes in brain parenchyma is observed up to 7 days after ischemia [110]. Over time, anti-inflammatory macrophages begin to dominate damaged brain parenchyma because they are necessary for parenchyma regeneration processes [110]. Other cells involved in the innate immune response triggered by brain ischemia are neutrophils which appear in injured brain tissue immediately after ischemia [110]. They concentrate close to the ischemic region and release oxygen free radicals, proinflammatory cytokines and proteolytic enzymes which cause additional destruction of brain tissue [110]. The number of neutrophils appearing in the brain after ischemia directly corresponds to the size of the ischemic injury region [110]. Furthermore, T and B lymphocytes infiltrate brain tissue postischemia. Brain ischemia also triggers penetration of brain parenchyma by natural killer cells [110]. Dendritic cells are involved in the immune response following brain ischemia as well [110]. Mast cells present in brain vessels and meninges also participate in the neuroinflammatory response following brain ischemia. Mast cells secrete cytoplasmic granules containing heparin, histamine, TNF-α and proteases, e.g., tryptase, chymase, matrix metalloproteinase-2 and matrix metalloproteinase-9, contributing to additional injury to the blood-brain barrier, brain edema and neutrophil infiltration in damaged brain parenchyma [110].

Lesions in white matter and neuroglial cell proliferation were noted in the brains of animals and humans following an episode of brain ischemia with reperfusion [18,19,22,23,63,64,67,68,111,112]. Ischemic injury of the brain in animals causes severe damage of the corpus callosum and subcortical white matter [19,63,64,113]. These observations are consistent with proliferation of neuroglial cells in both regions following ischemia-reperfusion brain injury [114]. Ischemia with reperfusion of the brain increases the permeability of the blood-brain barrier, which allows inflammatory cells with inflammatory mediators and amyloid to penetrate from the blood to the brain parenchyma, which in turn causes massive lesions in white matter [26,66,88,105,106,109,115]. 

Evidence suggests that transient brain ischemia with reperfusion in animals and humans causes massive loss of neuronal cells in the regions either belonging, or not, to areas of the brain selectively sensitive to an ischemic episode [14,18,19]. Progressive processes that continue during reperfusion after an ischemic episode are involved in ischemic brain neurodegeneration [14,19]. These processes develop not only in the early stages following brain ischemia, but also in the late periods after the resumption of brain circulation [14]. Over the years postischemia, developing neuropathological processes cause generalized brain atrophy [14,17,18,19,21]. A general brain examination performed following brain injury due to ischemia-reperfusion with survival up to 2 years showed hallmarks of brain hydrocephalus [17,18,19,21]. Dilatation of the subarachnoid space around the brain hemispheres has also been noted [18]. Complete hippocampal and striatal atrophy was reported [18,19]. The brain cortex after ischemia in animals and humans was narrow, showing artificially increased neuronal density [14,18,19]. In addition, features of late brain parenchyma atrophy, such as diffuse changes in white matter in the form of cavitations and rarefaction, revealing advanced spongiosis, have also been observed [18,19]. This phenomenon can be explained by a massive loss of neuronal cells, which is accompanied by an increased permeability of the blood-brain barrier occurring both in the early and late stages following ischemia-reperfusion brain injury [20,69,70,103]. 

## 9. The Development of Dementia Postischemia

After experimental brain ischemia injury, changes in behavior have been observed [9,10,11,12,73]. Locomotor hyperactivity was noted following ischemic brain damage, as in Alzheimer’s disease subjects, and is correlated with neuronal disappearance and development of neuroinflammation [20,22,23]. Postischemic brain damage causes the loss of reference and working memory with progress of a spatial memory deficit [9]. The progression of cognitive deficit develops slowly together with increase in length of recirculation time [9]. Long-lasting motor hyperactivity with cognitive deficits and decreased anxiety were also documented following repetitive transient postischemic brain injury in animals. The behavioral abnormality was associated with huge brain atrophy [17,18,19,21,67,68,74,75]. Learning and memory insufficiency following brain ischemia with recirculation move forward irreversibly and persevere forever [9].

Soon after outcome of ischemic neuropathology in humans, the slow and progressive development of dementia was found [15,116]. The occurrence of dementia following the first ischemic, and the recurrent, stroke is evaluated around 10% and 33–41%, respectively [116]. For the duration of a 25-year follow-up, the occurrence of dementia was estimated around 48% [116]. Worldwide, dementia following stroke occurs in between 5% and 50% of survivors, depending on diagnostic criteria, population demographics and geographical location [13]. In fact, it is certain that dementia following brain ischemia has many risk factors in common with the development of Alzheimer’s disease-type dementia. Among other things, reduced glucose metabolism in the brain is an early event in the pathology of Alzheimer’s disease and cerebral ischemia and may precede the neuropathological accumulation of amyloid in both disease entities [117,118]. In the progression of pathology, amyloid accumulation appears to play a key role, among other things, in the vessels of the brain, which again leads to a gradual decrease in cerebral blood flow postischemia, closing the vicious circle [118]. It is highly likely that after brain ischemia, alterations may precede Alzheimer’s disease dementia and cause all the consequences associated with the development of dementia in this disease entity. 

## 10. Discussion

**Figure 1:**
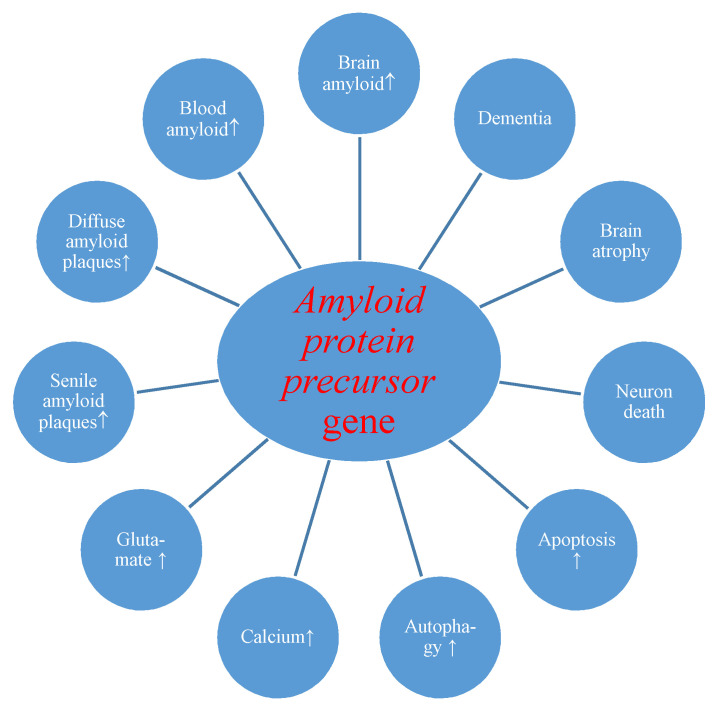
Potential role of amyloid protein precursor gene changes during brain injury due to ischemia-reperfusion. ↑-increase.

First of all, we present the reaction of the amyloid protein precursor gene and its product to transient brain ischemia with reperfusion. Evidence revealed postischemic overexpression of the *amyloid protein precursor* gene which correlated with the substantial rise of amyloid in the intra- and extracellular spaces of the brain [24,60] and serum [26,88,109] with generation of diffuse and senile amyloid plaques (Figure 1) [24,72]. 

**Figure 2:**
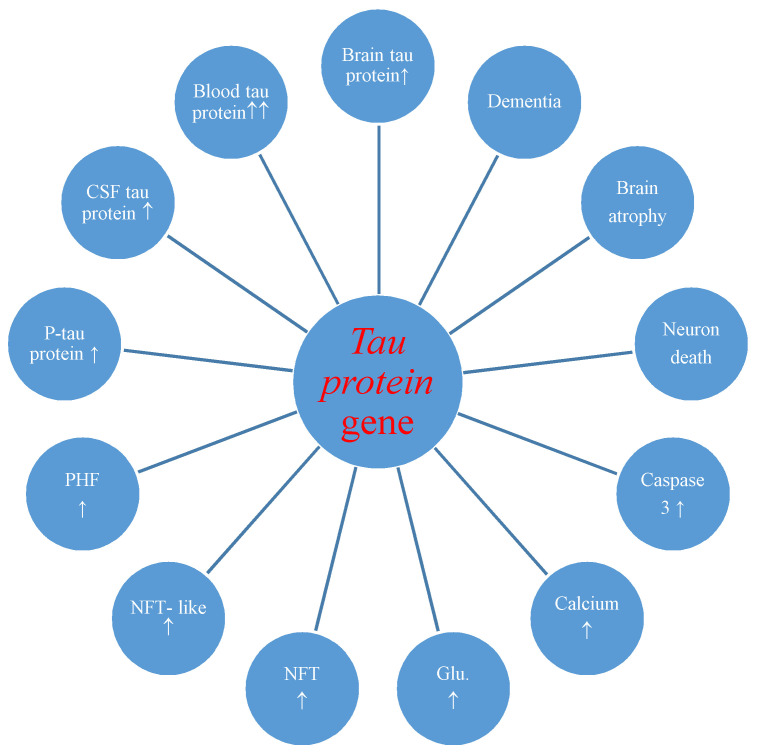
Potential role of tau protein gene changes during brain injury due to ischemia-reperfusion. CSF-cerebrospinal fluid, P-tau-phosphorylated tau protein, PHF-paired helical filaments, NFT-like-neurofibrillary tangle-like, NFT-neurofibrillary tangle, Glu.-glutamate, ↑-increase.

The studies also made known postischemic overexpression of the tau protein gene in the brain tissue which correlated with the increase of tau protein in the intra- and extracellular spaces [36] and plasma [29,30] with development of neurofibrillary tangles (Figure 2) [27]. 

Overexpression of the amyloid and tau protein genes begins at the same times as neuronal death and neurodegeneration postischemia (Figure 1 and Figure 2) [18,19]. Increased amyloid in brain parenchyma and blood [24,26,60,88,109] was correlated with a parallel growth of tau protein in brain tissue and plasma postischemia [29,30,36], and these alterations forecast a poorer clinical outcome. Postischemic tau protein gene overexpression also paralleled overexpression of the caspase 3 gene, which plays a significant role in apoptosis of neurons [91,93,94]. Additionally, it was noted that stimulated caspase shows a relationship with the occurrence of a neurofibrillary tangle (Figure 2) [36]. Also, postischemic neurodegeneration and dementia showed a negative relationship with the amount of amyloid and tau protein (Figure 1 and Figure 2) [19,36]. Presented facts indicate that neuronal injury and death in the postischemic brain need amyloid and tau protein. Therefore a new manner to control neuronal survival or death is presented (Figure 1 and Figure 2). Triggered neuropathological alterations, such as excitotoxicity, oxidative stress, autophagy, mitophagy, apoptosis and neuroinflammation through amyloid and tau protein clarify their probable neuropathological machinery in postischemic neurodegeneration (Figure 1 and Figure 2). Thus, it is highly likely that amyloid and tau protein, in addition, increase postischemic injury or neuronal death (Figure 1 and Figure 2).

## 11. Conclusions

Evidence points to proteomic and genomic alterations of amyloid and tau protein in the postischemic brain (Figure 1 and Figure 2). As a consequence, bilateral injury to the brain triggers postischemic neurodegeneration with development of dementia of an Alzheimer’s disease phenotype. Even so, a considerable move forward has, in recent times, been completed in research of the neuropathogenecity of amyloid and tau protein postischemia. However, strategic processes engaged in irreparable ischemic neurodegeneration produced through both proteins (Figure 1 and Figure 2) are, in spite of everything, unknown. In this way, animal reversible models of brain ischemia seem to be a helpful approach for clarifying the role of genes and their proteins straightforwardly connected with Alzheimer’s disease. With detailed study, the genomic and proteomic processes can speed up the existing knowledge about the neuropathogenesis of the postischemic brain, and stimulate upcoming exploration on brain ischemia with innovative trends. 

## Figures and Tables

**Table 1:** Changes in the expression of the Alzheimer’s disease-associated amyloid protein precursor gene in different brain structures at different times after experimental brain ischemia.

	Survival	2 Days	7 Days	30 Days
Structures				
CA1		**↓**	**↑**	**↑**
CA3		**↑**	**↑**	**↑**
MTC		**↓**	**↑**	**↑**

Expression: ↑ increase; ↓ decrease. CA1-CA1 area of hippocampus, CA3-CA3 area of hippocampus, MTC-medial temporal cortex.

**Table 2:** Changes in the expression of the Alzheimer’s disease-associated β-secretase gene in different brain structures at different times after experimental brain ischemia.

	Survival	2 Days	7 Days	30 Days
Structures				
CA1		**↑**	**↑**	**↓**
CA3		**↓**	**↓**	**↑**
MTC		**↑**	**↓**	**↓**

Expression: ↑ increase; ↓ decrease. CA1-CA1 area of hippocampus, CA3-CA3 area of hippocampus, MTC-medial temporal cortex.

**Table 3:** Changes in the expression of the Alzheimer’s disease-associated presenilin 1 gene in different brain structures at different times after experimental brain ischemia.

	Survival	2 Days	7 Days	30 Days
Structures				
CA1		**↑**	**↑**	**↓**
CA3		**↑**	**↑**	**↓**
MTC		**↔**	**↔**	**↔**

Expression: ↑ increase; ↓ decrease; ↔ no changes. CA1-CA1 area of hippocampus, CA3-CA3 area of hippocampus, MTC-medial temporal cortex.

**Table 4:** Changes in the expression of the Alzheimer’s disease-associated presenilin 2 gene in different brain structures at different times after experimental brain ischemia.

	Survival	2 Days	7 Days	30 Days
Structures				
CA1		**↑**	**↑**	**↓**
CA3		**↓**	**↓**	**↑**
MTC		**↑**	**↔**	**↔**

Expression: ↑ increase; ↓ decrease; ↔ no changes. CA1-CA1 area of hippocampus, CA3-CA3 area of hippocampus, MTC-medial temporal cortex.

**Table 5:** Changes in the expression of the Alzheimer’s disease-associated α-secretase gene in the CA3 of the hippocampus at different times after experimental brain ischemia.

	Survival	2 Days	7 Days	30 Days
Structures				
CA3		**↓**	**↓**	**↓**

Expression: ↓ decrease.

**Table 6:** Changes in the expression of the Alzheimer’s disease-associated tau protein gene in different brain structures at different times after experimental brain ischemia.

	Survival	2 Days	7 Days	30 Days
Structures				
CA1		**↑**	**↓**	**↓**
CA3		**↓**	**↑**	**↑**

Expression: ↑ increase; ↓ decrease. CA1-CA1 area of hippocampus, CA3-CA3 area of hippocampus.
